# Supplementary material for: Unlocking history through automated virtual unfolding of sealed documents imaged by X-ray microtomography
Source: Nat Commun. 2021 Mar 2;12:1184. doi: 10.1038/s41467-021-21326-w (PMC7925573; doi:10.1038/s41467-021-21326-w)
Supplement: Supplementary file 3 — Description of Additional Supplementary Files [file 41467_2021_21326_MOESM3_ESM.pdf]

# Description of Additional Supplementary Files

## File Name: Supplementary Movie 1

**Description:** An animation showing a physics-based unfolding simulation of the final reconstruction of DB-1538.

## File Name: Supplementary Foldable 1

**Description:** Letterlocking foldable of DB-1538, marked with mountain (red) and valley (blue) folding lines. Readers can print the model for themselves and fold into a model of the letterpacket. In its folded state, the front side of the model shows the letterpacket's address panel, and the back side shows the transparent view through the volumetric XMT data. The model references figure numbers in the main text. For a step-by-step guide of the folding order for the model, see Fig. 1 in the main text.

## File Name: Supplementary Foldable 2

**Description:** Letterlocking foldable of DB-1627, marked with mountain (red) and valley (blue) folding lines. Readers can print the model for themselves and fold into a model of the letterpacket. In its folded state, the front side of the model shows the letterpacket's address panel, and the back side shows the transparent view through the volumetric XMT data. The rest of the packet shows the reconstructed texture. The model references figure numbers in the main text. For a step-by-step guide of the folding order for the model, see Fig. 1 in the main text.
