# Supplementary figures and images for: Unlocking history through automated virtual unfolding of sealed documents imaged by X-ray microtomography
Source: Nat Commun. 2021 Mar 2;12:1184. doi: 10.1038/s41467-021-21326-w (PMC7925573; doi:10.1038/s41467-021-21326-w)

8d

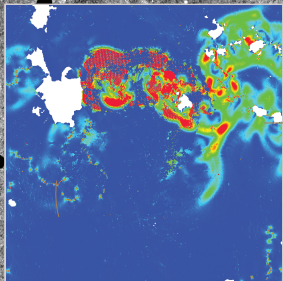

8a

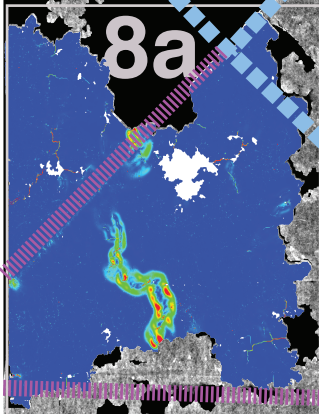

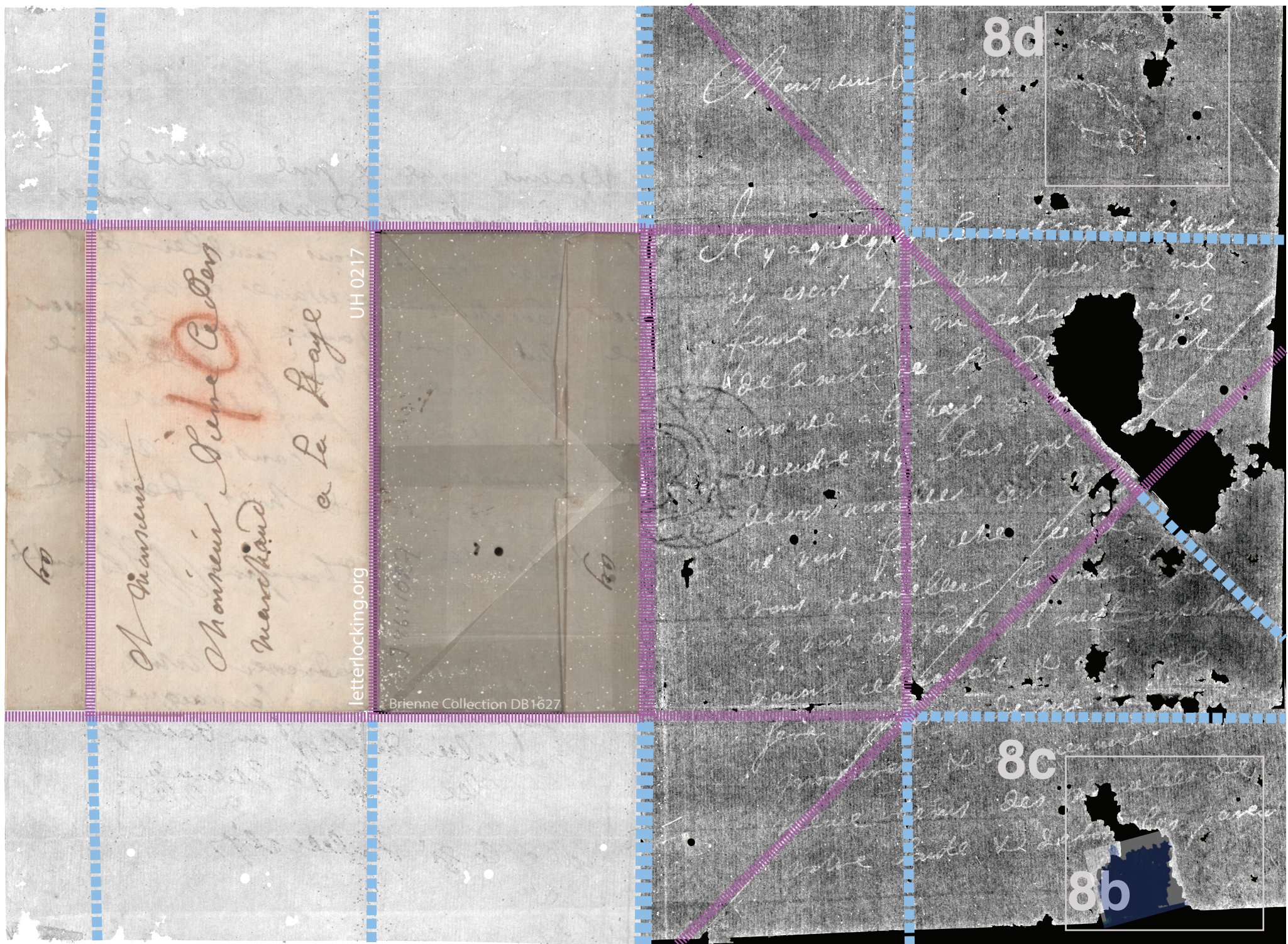

8d

UH 0217

letterlocking.org

Brienne Collection DB1627

8c

8b

Supplement: Supplementary file 5 — Supplementary Data [file 41467_2021_21326_MOESM5_ESM.zip › 248751_3_supp_5110818_qk9qwd.pdf]
