# Supplementary material for: Unlocking history through automated virtual unfolding of sealed documents imaged by X-ray microtomography
Source: Nat Commun. 2021 Mar 2;12:1184. doi: 10.1038/s41467-021-21326-w (PMC7925573; doi:10.1038/s41467-021-21326-w)
Supplement: Supplementary file 1 — Supplementary Information [file 41467_2021_21326_MOESM1_ESM.pdf]

SUPPLEMENTARY INFORMATION

Unlocking history through automated virtual unfolding of sealed documents imaged by X-ray microtomography

Dambrogio *et al.*

SUPPLEMENTARY NOTE 1

Images, transcription, and translation of virtually unfolded letter DB-1627

This supplement includes a transcription and translation of DB-1627, written to a French merchant, Pierre le Pers, assumed to be in The Hague. The letter was written by his cousin Jacques Sennacques in Lille, France, and sent on July 31, 1697. The transcription is based on the image in **Supplementary Figure 2** below. As historians and paleographers, who have collectively examined tens of thousands of early modern documents in all kinds of states and forms, we can confirm that the quality of the resulting image is no worse than some of the microfilms we have strained our eyes on, and in some ways it is better (due to the depth of the data). The amount of textual content that we have been able to extract is on par with letters we have seen in a physical form, for the idiosyncrasies of a hand must always be learned. Paleographers must rely on contextual clues and linguistic knowledge to fill in the blanks when text is messily written or actually missing. Some missing text in this particular letter is due to wormholes; in a few cases, primarily on the first page, it is unclear whether physical holes or incomplete algorithmic meshing accounts for the loss.

The letter is part of an archive of letters preserved by the chief postmasters of The Hague, a married couple called Simone de Brienne (*d.1707*) and Marie Germain (*d.1703*) [**1**]. Letters that could not be delivered were often kept by the Briennes' postal employees, since they represented a potential source of income: in the seventeenth and eighteenth centuries, the recipient rather than the sender paid for the letter. An undelivered letter could yet bring in some money if someone turned up to claim it. Each letter in this collection therefore received a mark in red crayon indicating how many *stuivers* (a Dutch coin) it would cost to receive it. The postal employees in The Hague often made further annotations on the letters providing the date of attempted delivery or the reason that the letter could not be delivered.

For example, letter DB-1627 bears the mark "10" in red crayon on the address panel, indicating that the recipient would need to pay 10 *stuivers*. The price on the letter provides an important clue as to the letter's origin: while we admittedly had some difficulty in deciphering the sender's city due to his handwriting, our guess of Lille is confirmed by the cost, as letters from the region of Lille were always priced at 10 *stuivers*, not to mention the fact that Lille possesses a rue St Etienne (a line that is clearly legible). A further annotation, the "ag" on the recto of the address panel, tells us that the letter arrived in The Hague in August, which seems logical given that it was sent from Lille on 31 July.

The content of this letter is typical for a family business letter of its time, though some expected formalities are not included at the beginning due to familial relationship, urgency, and the fact that this is the second time Sennacques has attempted to contact Le Pers. Sennacques, a legal professional in Lille, immediately requires a "legalized" (i.e. official, notarized) death certificate for a relation, Daniel Le Pers, perhaps due to a question of inheritance. His request issued, Sennacques then spends the rest of the letter asking for news of the family and commending his cousin to the graces of God. We do not know exactly why Le Pers did not receive Sennacques' letter, but given the itinerancy of merchants, it is likely that LePers had moved on.

Together, the letters in the Brienne Collection cast light on the everyday correspondence of a fascinating variety of individuals from all walks of life. They also bear witness to the fragility of lines of communication at a time when Europe was torn by war, economic crisis, and religious differences. People moved frequently, sometimes in haste, leaving no forwarding address because they did not have one, or they were not at liberty to divulge it.

A catalogue of the Brienne Collection is in preparation together with Early Modern Letters Online, an online union catalogue of early modern correspondence [**2**]. This is the first cataloguing project to adopt the letterlocking terminology of Jana Dambrogio and Daniel Starza Smith, as outlined in this article and **Supplementary Note 2**.

Transcription and translation of DB-1627

The following is a diplomatic transcription: this means that the transcription is rendered as faithfully as possible. Original line breaks are preserved. The often idiosyncratic spelling has not been modernized, early modern

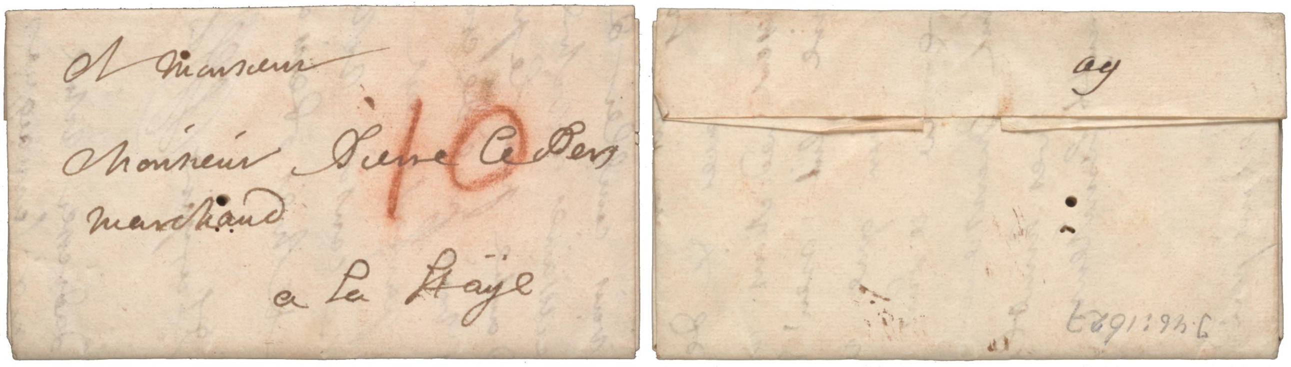

(a) Front of letterpacket. (b) Back of letterpacket.

Supplementary Figure 1: DB-1627 as unopened letterpacket.

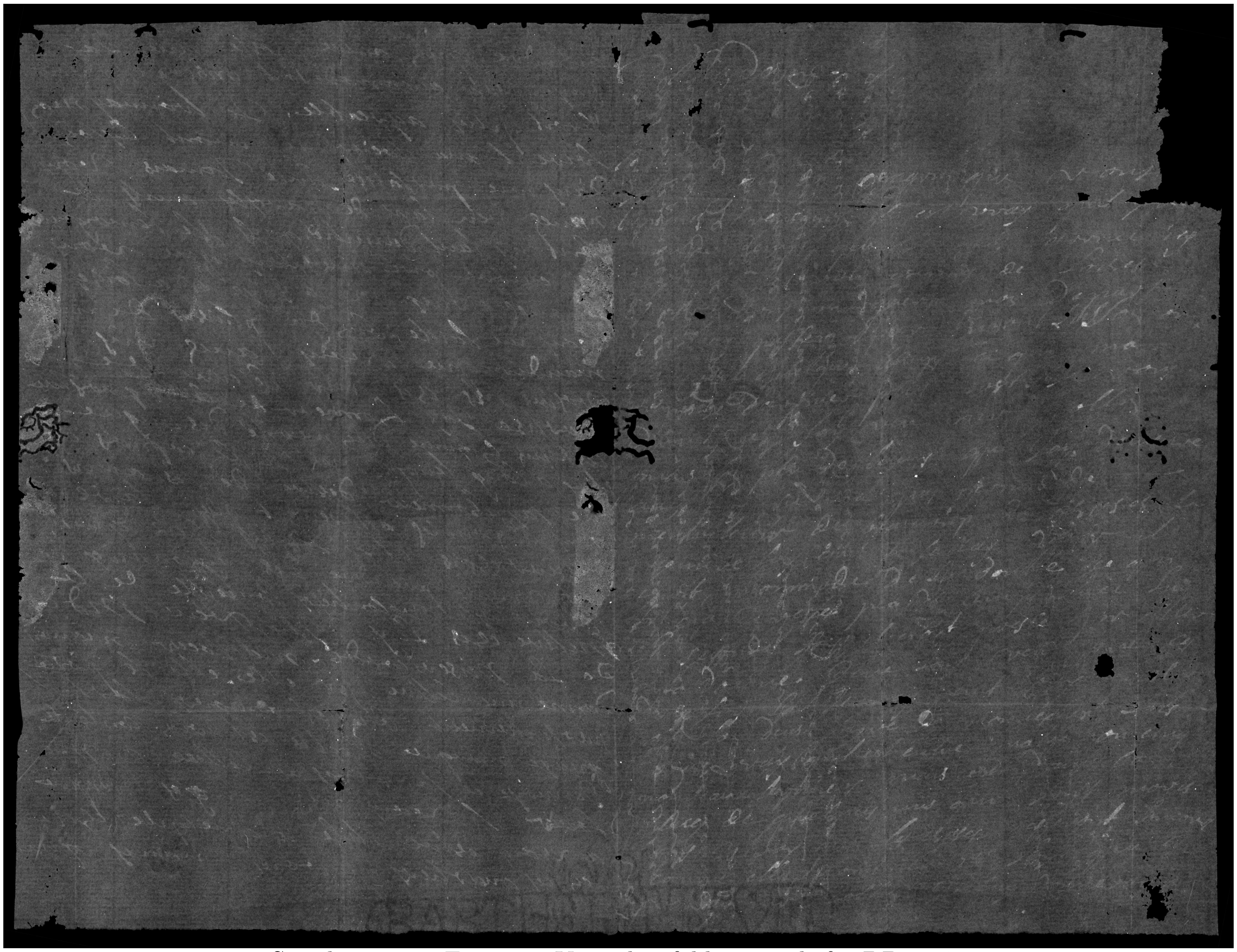

Supplementary Figure 2: Virtual unfolding result for DB-1627.

punctuation has been maintained. Abbreviations have not been expanded (e.g. “Mr” and “St” for “monsieur” and “saint”), and superscript letters are in superscript type (i.e. superscript). Letters or words that have been inferred are given between [square brackets]. In two cases, gaps in the algorithmically generated mesh resulted in complete textual loss; we estimate that approximately 10 characters are missing in each line, given

the average line length of this letter writer (30 characters). These instances are marked as {mesh hole}.The English translation attempts to strike a balance between the style of the author and intelligibility for today's reader. Punctuation has therefore been modernized and line breaks omitted.

[Letterpacket address panel, recto]

A Monsieur
Monsieur Pierre le Pers
marchand
a La Haÿe
10

[Letterpacket reverse panel, verso]

ag

[fol. 1r]

Monsieur & cousin
Il ỳ a quelque semaines que ie vous
aỳ escrit pour voùs prier de me
faire auoir vn extrait [leg]alizé
de la mort du Sr Da[niel le] Pers
arriuee a la haÿe au m[ois d]e
decembre 1695 sans que {mesh hole}
de vos nouvelles c'est le {mesh hole}
ie vous fais ceste seconde [pour]
vous renouueller la peine [que]
ie vous aỳ faite il mest important
d'auoir cet extrait & [vous] me
feriez plaisir de me [le faire]
procurer & de menuoier au
meme tems des n[o]uuelles de
votre sante & de tou[s] les parens

[fol. 2r]

& aussi que ie prie leternel de
vous continuer dans Ses Saintes
graces & de voùs combler des
benedictions necessaires de votre
salut rien d autre pour le present
sinon que ie vous prie de croire
[q]ue ie suis parfaitement
monsieur & cousin votre
tres humble & tres obeissant
seruiteur
Jacques JS Sen[n]a[cqu]es

Je vous prie dadresser votre
response a Mr Sennacques
conseiller du Roy au bailliage
[de Li]lle ruë St Etienne
a lille
[a lille] le 31 Juillet 1697

English translation of DB-1627

[Letterpacket address panel, recto]

To Monsieur Pierre le Pers, merchant in The Hague
10

[Letterpacket reverse panel, verso]

August

[fol. 1r]

Dear sir & cousin,

It has been a few weeks since I wrote to you in order to ask you to have drawn up for me a legalized excerpt of the death of sieur Daniel Le Pers, which took place in The Hague in the month of December 1695, without hearing from you. This is {...} I am writing to you a second time in order to remind you of the pains that I took on your behalf. It is important to me to have this extract you will do me a great pleasure to procure it for me to send me at the same time news of your health of all the family.

[fol. 2r]

I also pray that God maintains you in His Sainted graces & covers you with the blessings necessary to your salvation. Nothing more for the time being, except that I pray you to believe that I am completely, sir and cousin, your most humble & very obedient servant,

Jacques Sennacques

I beg you to send your response to Mr Sennacques, king's councillor in the bailiwick of Lille, Rue St Étienne in Lille
From Lille, the 31st of July 1697.

SUPPLEMENTARY NOTE 2
Glossary of letterlocking terms

The following definitions of letterlocking technical terms are derived from the Dictionary of Letterlocking (DoLL), a continually growing database available online and in preparation for print.

Assigned crease pattern

An **assigned crease pattern**, also known as an assigned mountain–valley crease pattern, is a designation of how each **crease** in a **letter** was created. It maps out the direction of each crease, in order to facilitate **reverse-engineering**. It is determined by combining a **crease pattern** with **crease assignment**. A crease pattern is the crisscross pattern of creases left in an unfolded writing **substrate**, indicating where **folds** were made. A crease assignment recognizes the direction of any individual original fold, either **mountain** or **valley**. If a substrate is turned over, crease assignment reverses.

Letterlocking

The act of folding and securing an epistolary writing **substrate** (such as papyrus, parchment, or paper) to function as its **envelope** or sending device. As distinct from the use of a **wrapper** or gummed envelope, and from **paper-folding traditions** such as origami. A sub-category of a 10,000-year **document security** tradition, pertaining to epistolary materials. The discipline which studies the materially engineered security and privacy of letters, both as a technology and a historically evolving tradition.

Letterlocking category (LC)

A letterlocking category is a distinct class of locked letters defined by the unique combination of physical **manipulations** used to transform a writing substrate into a **letterpacket**. Letterlocking category numbers (LCs) are assigned based on which manipulations appear to have been applied to the letterpacket. The letterlocking categories chart (see main article, **Fig. 7**) shows the 64 categories, based on all possible manipulation combinations. Unlocking History has assigned a category number to each historic letterpacket we have encountered, but we have not encountered examples of all categories. In the chart, block numbers indicate that historic examples have been encountered, while outline numbers represent hypotheticals. Categories record only the presence of a manipulation, rather than dictating the order by which they are applied, or whether they are applied singularly or in multiples. A single category can encompass many category variations. Category information is used to help determine **security score**.

Letterlocking configuration

The unique manifestation of any single locked letter.

Letterlocking format (LF)

A letterlocking format is a distinct class of locked letters defined by the number of **edges** on the two-dimensional silhouette of a **letterpacket**. Format is a product of a letterpacket's **internal engineering**, and can be determined by studying an opened letter's **assigned crease pattern**. Letterlocking format numbers (LFs) are assigned based on edge-number and, in some cases, features of orientation or shape. This is especially important for packets with four edges, which are the most common; these are sub-divided into squares, diamonds, and rectangles. Rectangles are further defined as wide or tall depending on how they must be held in order to read the address. Extra-wide and extra-tall formats also exist.

The letterlocking formats chart shows 18 formats (see main article, **Fig. 7**). This number is representative rather than exhaustive, since a letterpacket could conceivably have dozens or hundreds of edges depending on the intricacy of folding. Some letters can be rolled into scrolls or spheres, shapes without edges; these are counted as having infinite edges. Unlocking History has assigned a format number to each historic letterpacket we have encountered, but we have not encountered formats with 11 edges or more than 12 edges.

Letterlocking manipulations A physical alteration performed on a writing **substrate** in order to turn it into a **letterpacket** and secure it shut. **Manipulations** include:

- **fold/roll**
- **tuck**
- **adhere**
- **slit/hole**
- **lock** – further subdivided into **lock-O**, **lock-SA**, and **lock-SU**

Every letterlocking category is characterized by a specific combination of manipulations: the more manipulations used, the higher the **letterlocking category** assigned to the letterpacket.

1. *Fold.* The doubling or bending of a substrate over itself. Most locked paper **letters** are folded into flat packets, but rolled packets also exist. A fold usually leaves a **crease** in the substrate (see **mountain crease** and **valley crease**).
2. *Slit/hole.* A slit is a linear piercing made in a substrate by a flat blade. A hole is a puncture made by an **awl**, a needle, or similar boring tool. Both provide space for **locks** to travel through a **substrate**, and are therefore treated as serving the same function, however a slit/hole that is not a part of the **locking mechanism** may be observed.
3. *Tuck.* The insertion of a flap into a pocket made by **folding** the **substrate**. The flap may consist of single or multiple layers. One of the principal **manipulations** of **letterlocking**.
4. *Adhere.* The application of an **adhesive** substance, such as **sealing wax** or a starch **wafer**, to affix substrates and/or lock surfaces to one another. One of the principal **manipulations** of letterlocking.
5. *Lock.* A material appendage to the main writing **substrate**, used to fasten or secure a **letterpacket** shut. As part of the **locking mechanism**, locks often (though not always) work in combination with **slits/holes** and/or **adhesive**. One of the principal manipulations of **letterlocking**. Locks are subdivided into three kinds: **Lock–Other** (lock-O), **Lock-Same Substrate**, **Attached** (lock-SA), and **Lock–Same Substrate**, **Unattached** (lock-SU).
   - *Lock–Other (lock-O).* A **lock** formed from a source other than the writing **substrate** of the **letter**. O signifies "other." Lock-O materials could include **floss**, **parchment**, **paper**, wire, or even hair.
   - *Lock–Same Substrate, Attached (lock-SA).* A **lock cut into** or **sliced** internally from the writing **substrate** of the **letter**, but not detached entirely before it is used to **secure** the **letterpacket** shut. SA signifies "same substrate, attached."
   - *Lock–Same Substrate, Unattached (lock-SU).* A **lock cut off** or **sliced** from the writing **substrate** of the **letter**, and detached entirely before it is used to **secure** the packet shut. SU signifies "same substrate, unattached."

We cannot know a historic letterlocker's intentions, so security scores are always based on the surviving material evidence alone. Security scores can be assigned to:

- an opened, partially opened, or closed letterpacket that preserves its locking evidence;
- an unopened letterpacket, by the locker themselves
- an unopened letterpacket, by someone who observed the **manipulations** as they were made
- an unopened letterpacket, scanned and digitally unfolded.

Security scores can also be inferred for unopened letterpackets not yet scanned or for opened letters missing some of their evidence.

SUPPLEMENTARY NOTE 3
Virtual unfolding results

From our virtual unfolding results, we can generate additional visualizations of the letters' folded geometry, such as physics-based simulations of the unfolding process (see **Supplementary Movie 1**) and printable models of the letterpackets (see **Supplementary Foldable 1** and **2**). Our foldable models are marked with mountain and valley folding lines, which readers can print for themselves and fold into a model of the letterpacket. In its folded state, the front side of the model shows the letterpacket's address panel, and the back side shows the transparent view through the volumetric XMT data. The rest of the packet is colored to match **Fig. 5** in the main text. More animations and foldables are presented on our supplementary website brienne.org/unfolding.

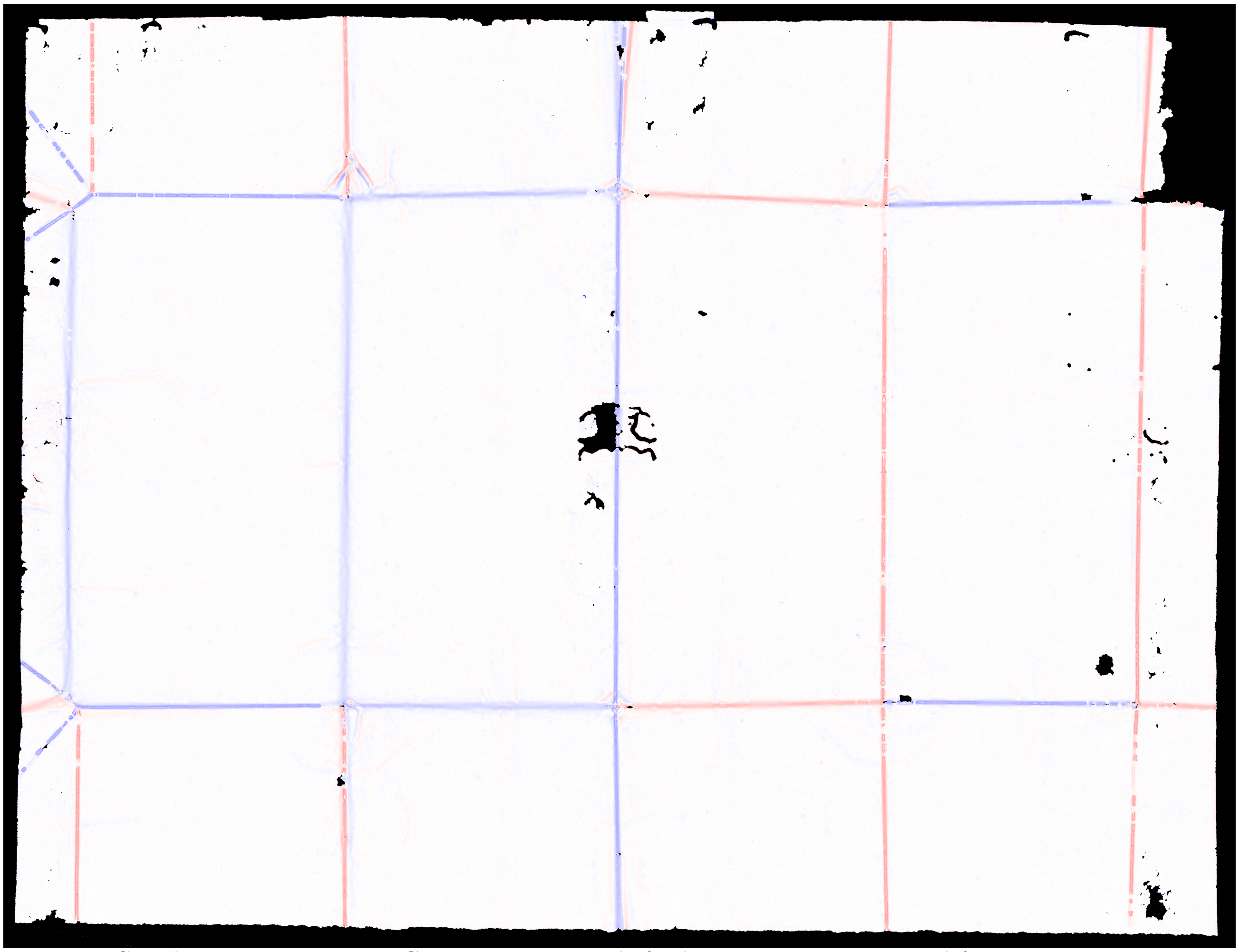

Supplementary Figure 3: Crease pattern result for largest section extracted from DB-1538.

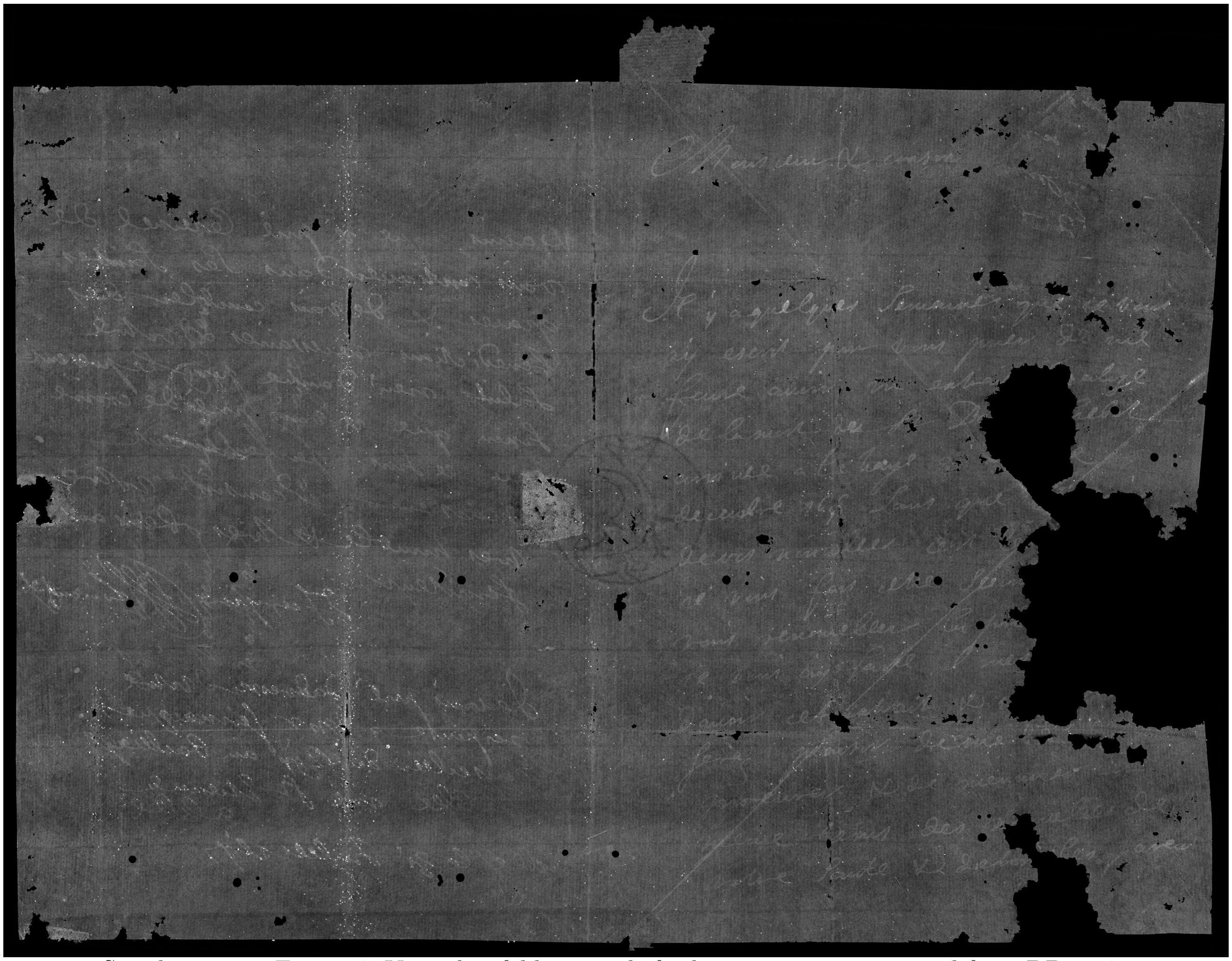

Supplementary Figure 4: Virtual unfolding result for largest section extracted from DB-1627.

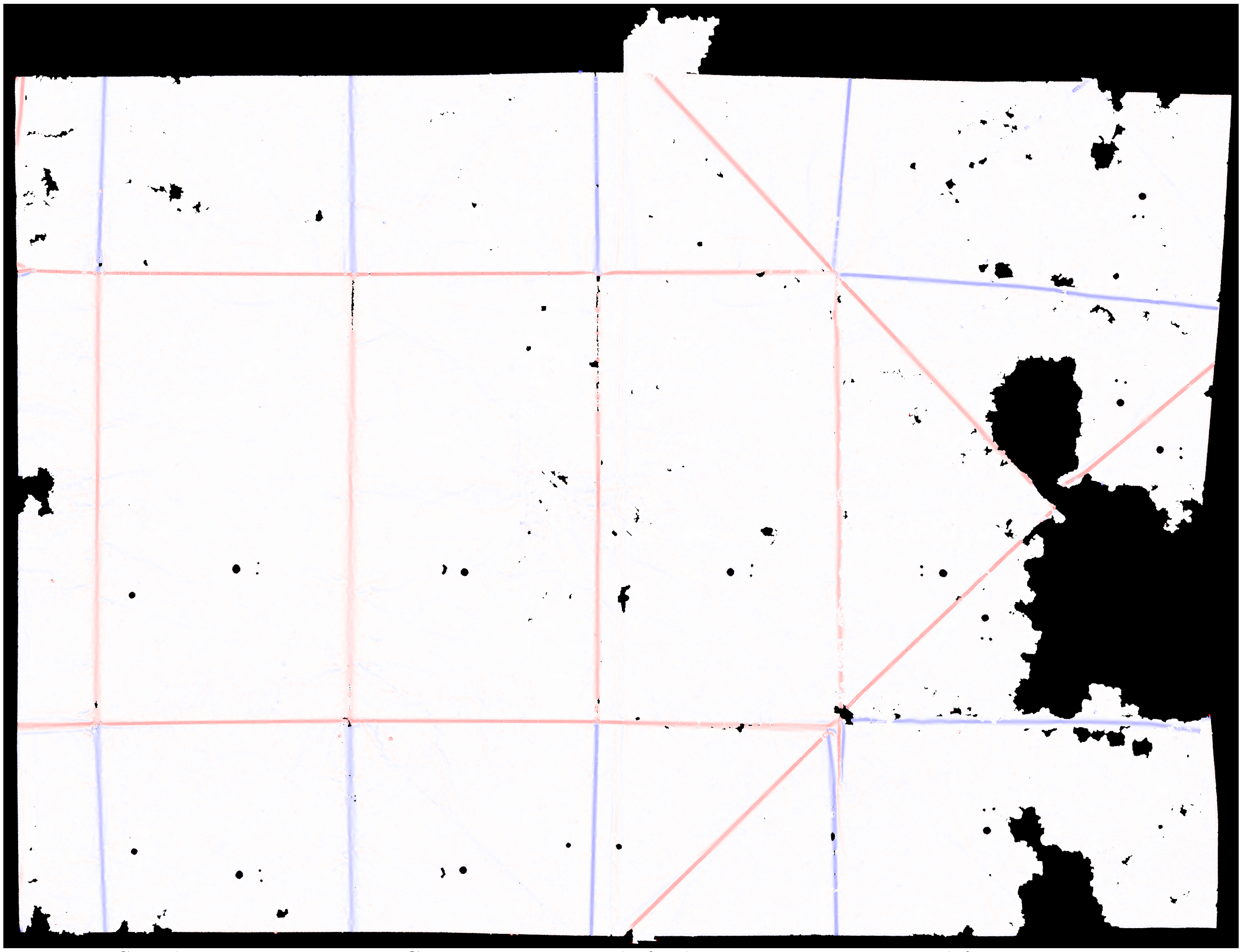

Supplementary Figure 5: Crease pattern result for largest section extracted from DB-1627.

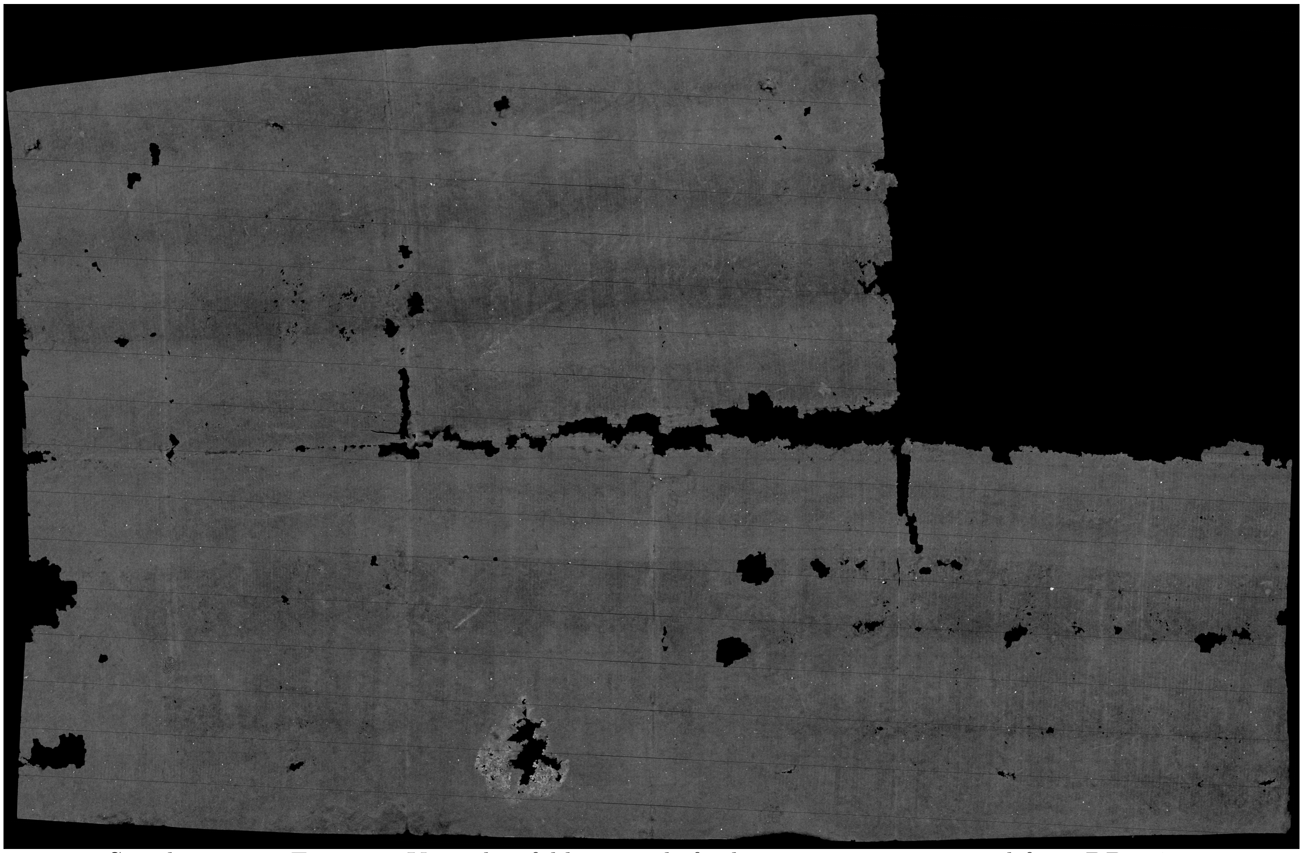

Supplementary Figure 6: Virtual unfolding result for largest section extracted from DB-1976.

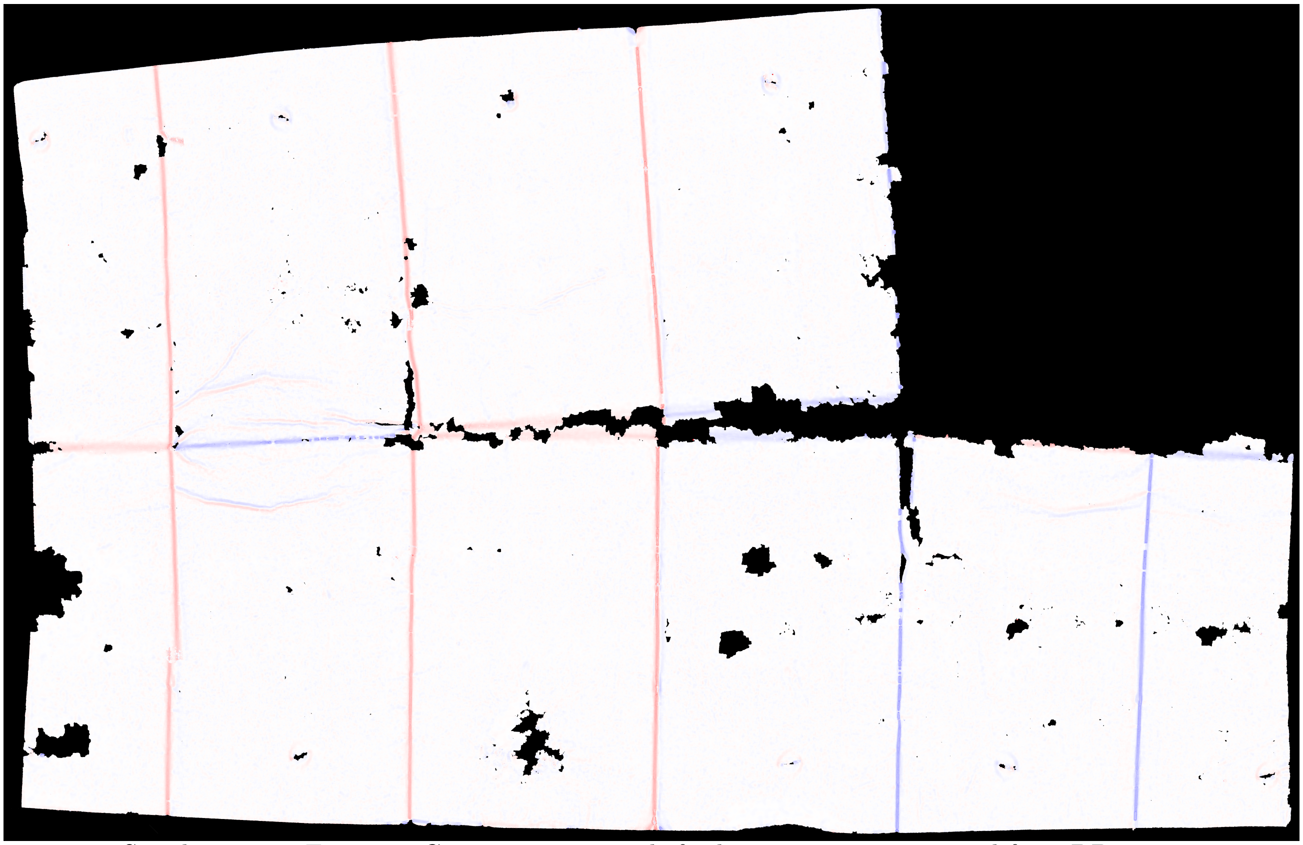

Supplementary Figure 7: Crease pattern result for largest section extracted from DB-1976.

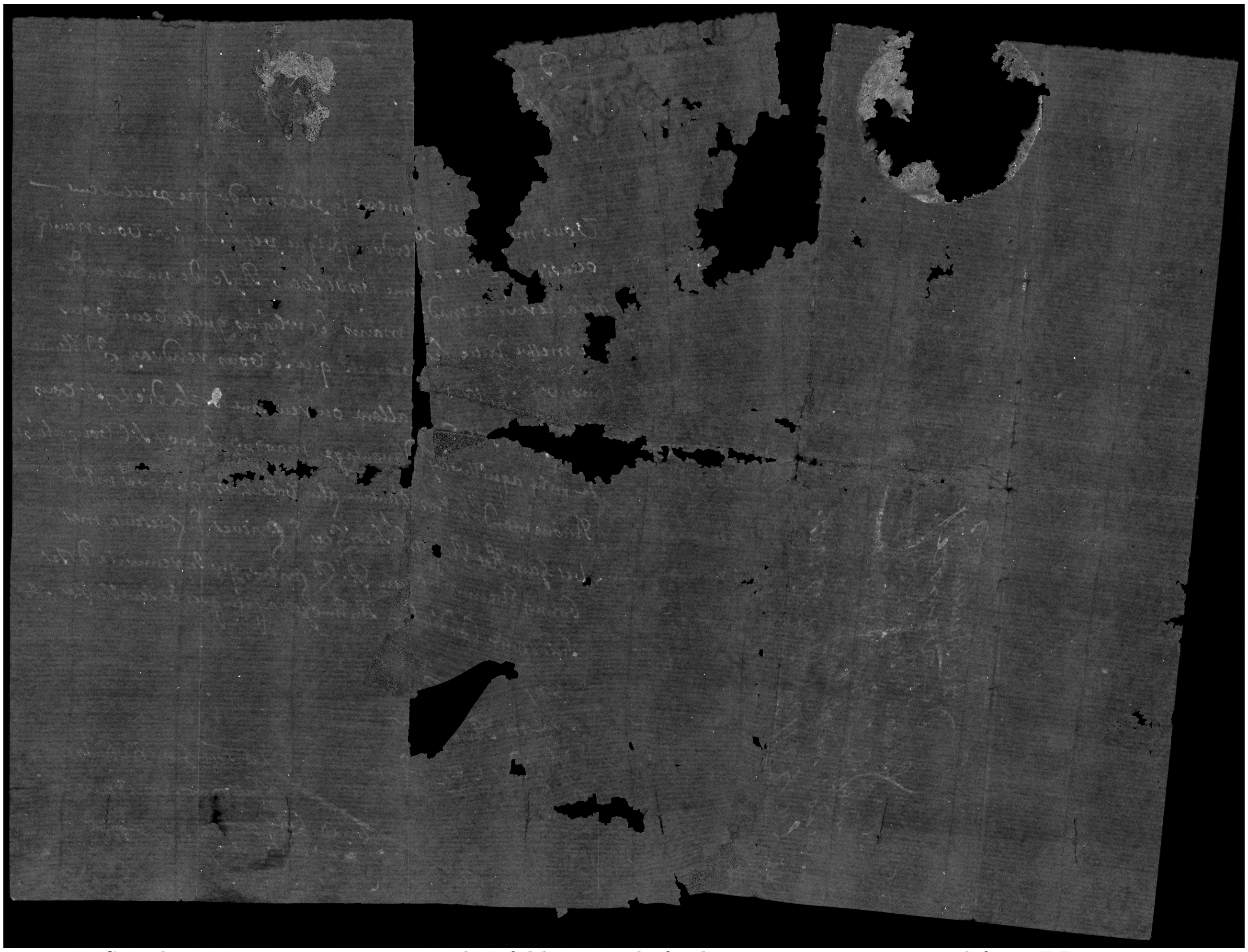

Supplementary Figure 8: Virtual unfolding result for largest section extracted from DB-2040.

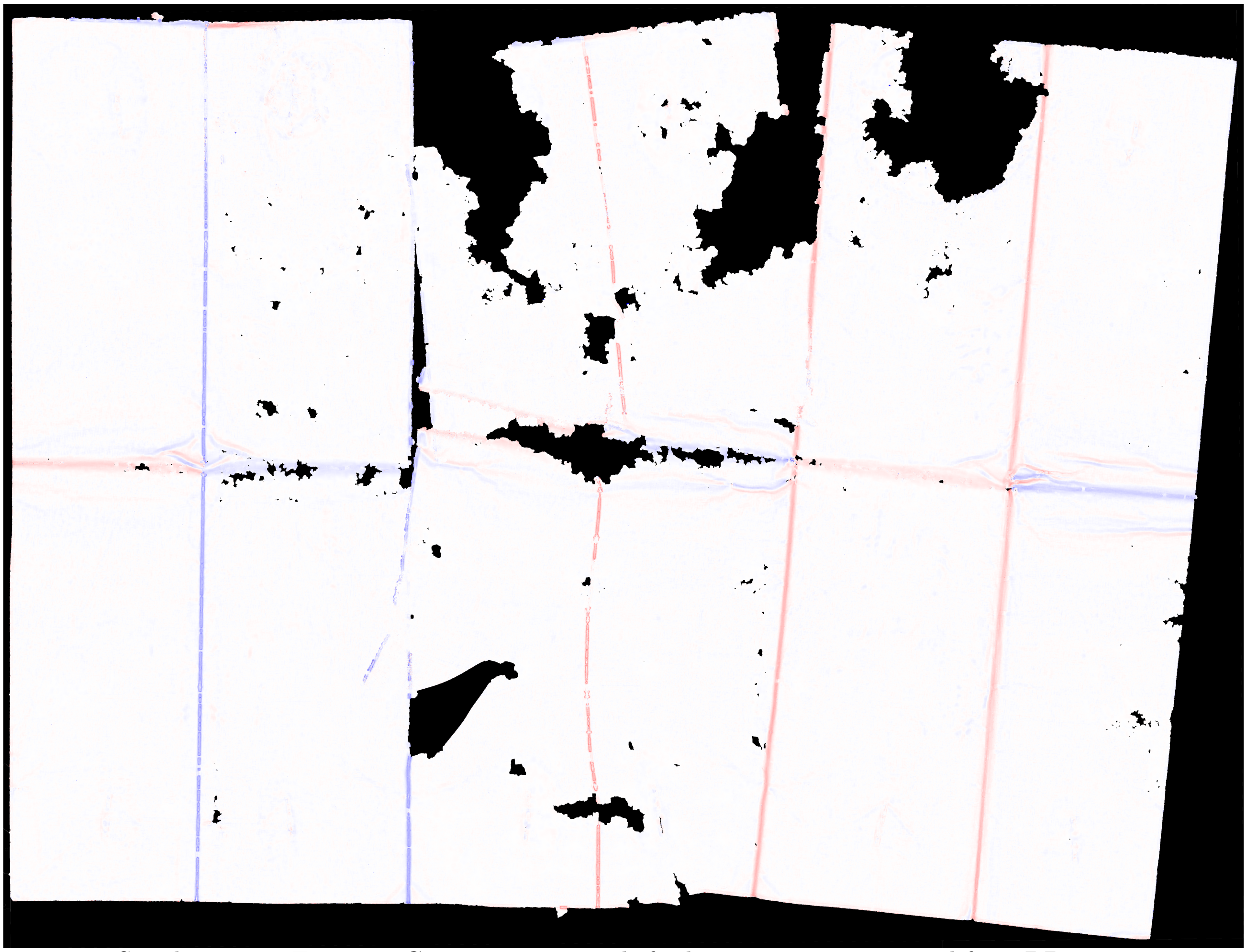

Supplementary Figure 9: Crease pattern result for largest section extracted from DB-2040.

SUPPLEMENTARY METHODS

Virtual unfolding methods

Volumetric Scanning

Eleven Brienne letters were imaged in a MuCAT2 scanner run by the microtomography research group at Queen Mary, University of London (QMUL). The letter-sets were chosen without lead-containing seals, which create severe artifacts. The letters were seated on one of their short edges, with minimal flex, and left to acclimatize overnight before scanning. Plastazote® low-density polyethylene spacers were inserted between the letters within the container so that each was easily discernible in the scanning data. The imaging field size was set to 2100 pixels with a further 32 pixels each side to record the incident intensity (dark and flat field images were also recorded). Scan time was just under three days. After the scan, a calibration carousel [**3**] was scanned to allow accurate beam-hardening correction. Tomographic reconstruction was performed using the Feldkamp back-projection algorithm [**4**] and all five volumes were concatenated to form a single 2030×2030×2970 voxel image.

The unique MuCAT2 system, designed and run at QMUL [**5**], uses a time-delay integration (TDI) readout moving camera (800-series) to obtain X-ray images that are wider than the static imaging field of the camera, have a high dynamic range (giving good contrast ratio), and yield reconstructed tomographic images free from ring artifacts. The voltage was set to 30 kV for optimal contrast and the current to 0.54 mA, which is on the power limit beyond which excessive pitting of the X-ray target would occur (pitting causes beam hardening issues and problems with the scan calibration). In order to scan the stack of letters within the maximum TDI imaging field, and within a reasonable time, the resolution was limited to give an effective voxel size of 38μm; the width of each paper layer measures about 2.5 voxels.

The raw CT data was scaled and quantized, resulting in a volumetric array of 8-bit, greyscale voxels representing material density in 3D space. Each letterpacket was cropped from the combined dataset and processed independently.

Segmentation

Our initial segmentation methods closely follow the methods described by Steger[**6**, **7**] to perform ridge detection in 2D and 3D image data. We repeat Steger's methods and notation here and extend these methods via normal relaxation, oriented blurring, and feature point filtering.

First, we calculate partial derivatives ($\mathbf{r}$) of the raw data ($\mathbf{R}$) with respect to the spatial dimensions x, y, and z for each voxel in the dataset by convolving $\mathbf{R}$ with a series of Gaussian derivative kernels ($\mathbf{G}$):

$$\begin{aligned} \mathbf{r}_x &= \mathbf{G'}_x * \mathbf{G}_y * \mathbf{G}_z * \mathbf{R} \\ \mathbf{r}_y &= \mathbf{G}_x * \mathbf{G'}_y * \mathbf{G}_z * \mathbf{R} \\ \mathbf{r}_z &= \mathbf{G}_x * \mathbf{G}_y * \mathbf{G'}_z * \mathbf{R} \end{aligned} \tag{1}$$

$$\begin{aligned} \mathbf{r}_{xy} &= \mathbf{G'}_x * \mathbf{G'}_y * \mathbf{G}_z * \mathbf{R} \\ \mathbf{r}_{yz} &= \mathbf{G}_x * \mathbf{G'}_y * \mathbf{G'}_z * \mathbf{R} \\ \mathbf{r}_{xz} &= \mathbf{G'}_x * \mathbf{G}_y * \mathbf{G'}_z * \mathbf{R} \end{aligned} \tag{2}$$

$$\begin{aligned} \mathbf{r}_{xx} &= \mathbf{G''}_x * \mathbf{G}_y * \mathbf{G}_z * \mathbf{R} \\ \mathbf{r}_{yy} &= \mathbf{G}_x * \mathbf{G''}_y * \mathbf{G}_z * \mathbf{R} \\ \mathbf{r}_{zz} &= \mathbf{G}_x * \mathbf{G}_y * \mathbf{G''}_z * \mathbf{R} \end{aligned} \tag{3}$$

where $*$ is the convolution operator and the subscript of the Gaussian smoothing kernel indicates its orientation.

Assuming the writing substrate has an expected cross sectional width (w_{exp}), we used the following parameters for $\mathbf{G}$, $\mathbf{G}'$, and $\mathbf{G}''$:

$$\sigma = \frac{w_{exp}}{2\sqrt{3}} = 0.7 \tag{4}$$

$$\mu = 0 \tag{5}$$

$$k = 3 \tag{6}$$

where σ is the standard deviation, μ is the mean, and k is the size of the kernels.

We use the partial derivatives to construct a Hessian matrix $\mathbf{H}$ for each voxel in the dataset:

$$\mathbf{H} = \begin{bmatrix} r_{xx} & r_{xy} & r_{xz} \\ r_{xy} & r_{yy} & r_{yz} \\ r_{xz} & r_{yz} & r_{zz} \end{bmatrix} \tag{7}$$

where r_{xx} is the scalar value of $\mathbf{r}_{xx}$ inside the current voxel.

Following Steger[**6**, **7**], the eigenvector corresponding to the eigenvalue of largest absolute value is normalized and designated as the normal vector for that voxel ($\hat{\mathbf{n}}$). The largest eigenvalue of $\mathbf{H}$ corresponds to the second derivative at that voxel; we call this the "response" and denote it by λ.

$\hat{\mathbf{n}}$ is susceptible to noise due to local variations in ink distribution and paper density. We expect that the direction of the surface normals should vary smoothly across the dataset, so we apply an iterative relaxation to the normals calculated at each voxel.

For each iteration of **normal relaxation**, we calculate a new normal for each voxel based on a weighted average of its current normal and the normals of its six axis-aligned neighbors. For a voxel at position x, y, z with normal vector $\hat{\mathbf{n}}_i$ and response λ_i, its new normal ($\hat{\mathbf{n}}_{i+1}$) and response (λ_{i+1}) is calculated by:

$$\hat{\mathbf{n}}_{i+1}(x,y,z) = \frac{|\lambda_i(x,y,z)| * \hat{\mathbf{n}}_i(x,y,z) + \sum_{neighbor} |\lambda_i(neighbor)| * \hat{\mathbf{n}}_i^*(neighbor)}{|\lambda_i(x,y,z)| + \sum_{neighbor} \lambda_i(neighbor)} \tag{8}$$

$$\lambda_{i+1} = r_{xx}n_{i+1,x}^2 + r_{yy}n_{i+1,y}^2 + r_{zz}n_{i+1,z}^2 + 2r_{xy}n_{i+1,x}n_{i+1,y} + 2r_{yz}n_{i+1,y}n_{i+1,z} + 2r_{xz}n_{i+1,x}n_{i+1,z} \tag{9}$$

where $n_{i+1,x}$ denotes the x component of $\hat{\mathbf{n}}_{i+1}$.

We found that a total of 40 iterations of normal relaxation was sufficient for our dataset.

We apply a **2D Gaussian blur** to the dataset, oriented with the surface of the paper to smooth out variations in the data caused by the presence/absence of ink, fluctuations in the density of the paper, and occlusions in the scan. We orient this smoothing at each voxel so that it is orthogonal to $\hat{\mathbf{n}}$. We found $\sigma_{blur} = 0.5$ and $k_{blur} = 1$ were appropriate for our dataset. The result after blurring is called $\mathbf{R}_{blur}$.

Following Steger, **feature point detection** assumes that the raw data is a discrete approximation to a continuous differentiable function. For each voxel, we can approximate $f(t)$, a continuous differentiable function along $\hat{\mathbf{n}}$, with the second-order Taylor expansion:

$$f(t) = R_{blur} + r't + \frac{1}{2}r''t^2 \tag{10}$$

where t is a spatial variable along $\hat{\mathbf{n}}$ (with $t = 0$ in the center of the voxel) and R_{blur}, r', and r'' are the zeroth, first, and second derivatives of $\mathbf{R}_{blur}$ with respect to t, evaluated inside the voxel. Evaluating the first derivative of $f(t)$ we have:

$$f'(t) = r' + r''t \tag{11}$$

Solving for $f'(t) = 0$ we get:

$$t = -\frac{r'}{r''} \tag{12}$$

We are interested in detecting points on the medial surface of the paper — these points correspond to a local maximum in density along each voxel's normal. By evaluating Equation 12 for each voxel in the dataset, we can determine the sub-voxel location of a local maximum or minimum of $f(t)$. We extract a strip of $\mathbf{R}_{blur}$ oriented along $\hat{\mathbf{n}}$ and convolve it with $\mathbf{G}'$ and $\mathbf{G}''$ to approximate r' and r''. The subvoxel position ($\mathbf{p}$) of the feature point associated with a given voxel is given by:

$$\mathbf{p} = t\hat{\mathbf{n}} \tag{13}$$

Feature points detected this way must meet some criteria in order to be declared valid points. First, the detected point must lie within the bounds of the current voxel (i.e. no component of $\mathbf{p}$ may be less than -0.5 or greater than 0.5). Following Wagner's implementation [**8**] of Steger's methods we relaxed this constraint slightly; instead we used -0.6/+0.6 as a cutoff.

$$[-0.6, -0.6, -0.6] \leq \mathbf{p} \leq [0.6, 0.6, 0.6] \tag{14}$$

For bright surfaces on a dark background, we expect the second derivative along the surface normal of the paper to be negative (concave down / local maximum):

$$\lambda < 0 \tag{15}$$

Additionally we require that R_{blur} is above a noise threshold (c_{noise}) of 25:

$$R_{blur} > c_{noise} \tag{16}$$

We calculate $\mathbf{e_3}$, the 3D absolute position of a feature point, by adding $\mathbf{p}$ to the absolute position of the center of the voxel at index (x, y, z):

$$\mathbf{e}_3 = \mathbf{p} + \begin{bmatrix} x + 0.5 \\ y + 0.5 \\ z + 0.5 \end{bmatrix} \tag{17}$$

We calculate the approximate **paper width** associated with each feature point by detecting the bounds of the paper along its surface normal. First we extract a strip of $\mathbf{R}_{blur}$ around each feature point, oriented along $\hat{\mathbf{n}}$. We use trilinear interpolation to calculate the value of the strip with subvoxel precision. We search along each strip in either direction until we find a location where the value of $\mathbf{R}_{blur}$ drops below c_{noise} or where there is a strong concave up curvature. We use these left and right bounds to calculate a paper width.

We compare paper width against a maximum expected paper width (w_{max}) to determine if a point lies on the medial surface of a single layer of paper or if it has been incorrectly placed between multiple layers of closely packed paper. Points exceeding $w_{max} = 6$ voxels are flagged; they will be re-evaluated later (Section).

The remaining feature points are connected by edges to form a mesh. Our **meshing** strategy follows directly from a similar implementation described by Steger[**7**], with some additional geometric constraints. First, we

query a one-voxel radius around each feature point to identify all its neighbors. If two feature points are within 0.5 voxels of each other, one of them is destroyed. We form edges between the remaining vertices, subject to the following criteria:

- The voxel normals ($\hat{\mathbf{n}}$) of any pair of vertices connected by an edge must be aligned, within $\pi/6$ radians
- All edges emanating from a vertex must be perpendicular to $\hat{\mathbf{n}}$, within $\pi/6$ radians
- No two edges emanating from the same vertex may have an included angle of less than $\pi/6$ radians
- No edge may have length greater than 1.75 voxels or less than 0.5 voxels

Depending on the quality of the CT data, paper width filtering may omit large regions of points, usually in areas of the CT scan where two or more paper layers are closely packed. Subsequently, the meshed surface generated by segmentation may contain holes or form many separate connected components; in fact, a typical letter from our dataset generates about 10,000-20,000 connected components.

We identify all the connected components and flag any that contain fewer than ten vertices. Additionally, we flag any vertices connected by fewer than three edges. We remove flagged vertices from our initial mesh.

Flattening

The mesh we have generated so far corresponds to the folded geometry of the letter packet in $\mathbb{R}^3$, which we call embedding E_3. We now compute E_2, a nearly isometric embedding of the mesh in $\mathbb{R}^2$ that corresponds to the paper's "unfolded" state. Starting from a single triangle mapped to the origin, we incrementally map mesh vertices from E_3 to E_2, subject to edge-distortion and orientation-preserving constraints. We add new vertices to the boundary of E_2, and run a distortion-minimizing step, based on minimizing the energy of a system of springs.

For the remainder of this document, we will use the following convention:

$$\text{vertex } i \text{ has position } \mathbf{e_{3,i}} \text{ in } E_3 \text{ (in } \mathbb{R}^3) \text{ and position } \mathbf{e_{2,i}} \text{ in } E_2 \text{ (in } \mathbb{R}^2)$$

For each vertex i, we identify all neighbors within a 2-ring neighborhood in E_2. We use the neighbors' positions in E_3 and E_2 to calculate a $\mathbf{e_{2,i}}$ that minimizes the L2 error (of squared distances, to avoid square roots):

$$\min_{\mathbf{e_{2,i}}} \sum_{neighbors} (\|\mathbf{e_{2,i}} - \mathbf{e_{2,neighbor}}\|^2 - \|\mathbf{e_{3,i}} - \mathbf{e_{3,neighbor}}\|^2)^2 \tag{18}$$

We use the Broyden-Fletcher-Goldfarb-Shanno (BFGS) algorithm to numerically evaluate an optimal $\mathbf{e_{2,i}}$.

In order to add a vertex to E_2, it must also satisfy the following conditions:

- Edges in E_2 may not be stretched or compressed more than 0.5 voxels from their length in E_3
- The relative winding order of neighboring triplets of vertices must be consistent between E_3 and E_2

After each new row of vertices is added to E_2, we use a physics-based approach to globally minimize edge length distortion. We model the flattened mesh as a mass-spring system by applying masses at its vertices and linear-elastic springs along each edge. We integrate the system forward by a fixed number of time steps to allow the edges of the mesh to move toward a minimally strained configuration.

In this section we have introduced a 2D embedding called E_2. In practice, E_3 may contain several connected components that cannot be mapped to a single E_2 with a globally consistent reference frame. Instead, we compute a separate E_2 for each connected component in E_3:

$$E_2 = \{E_{2,1}, E_{2,2}, \ldots, E_{2,k-1}, E_{2,k}\}$$

Each $E_{2,n}$ has its own reference frame in $\mathbb{R}^2$. In the following section, we compute rigid transformations between $E_{2,n}$ and $E_{2,m}$. For the remainder of this document we will refer to the connected components of E_3 as "components", or "mesh components".

Hybrid Mesh Propagation

Recall that, at this point, the mesh has many discontinuities and is split into many connected components. At this stage, E_3 typically covers about 50-80% of the folded letterpacket.

Vertex addition occurs on E_3. Beginning at vertices on the boundary of the mesh, we add new vertices inside neighboring voxels that satisfy:

- neighboring voxel does not already contain a mesh vertex
- neighboring voxel has $R_{blur} > c_{noise}$

From a given boundary vertex at position $\mathbf{e_{3,boundary}}$, we calculate a new vertex position ($\mathbf{e_{3,projected}}$) in a neighboring empty voxel by:

$$\mathbf{e_{3,projected}} = \mathbf{e_{3,voxel}} + \left[(\mathbf{e_{3,boundary}} - \mathbf{e_{3,voxel}}) \cdot \hat{\mathbf{n}}_{voxel}\right]\hat{\mathbf{n}}_{voxel} \tag{19}$$

where $\mathbf{e_{3,voxel}}$ is the center position of the empty voxel and $\hat{\mathbf{n}}_{voxel}$ is its associated (relaxed) normal vector. This is equivalent to projecting $\mathbf{a}_{voxel}$ on the plane defined by $\hat{\mathbf{n}}_{voxel}$ and $\mathbf{e_{3,boundary}}$.

We find all existing vertices within a one voxel radius of $\mathbf{e_{3,projected}}$. We mesh the projected vertex to its neighbors following the same criteria as those used during the meshing phase of segmentation. We analyze the connectivity of the neighbors to determine if the projected vertex is bridging two separate mesh components and only add the vertex to one of these components; this prevents separate components from attaching to each other at this stage of the algorithm. If the projected vertex meets the following criteria, it is added to the mesh:

- All vertices must form at least three edges with existing mesh
- No edges may have a length less than 0.5 voxels or greater than 1.75 voxels

After each round of additions in E_3, we add new vertices to E_2, following the procedure outlined in the previous section. We continue to run distortion minimization on E_2 throughout the mesh growing process.

We use a physics-based approach to perturb the new vertices' positions in E_3 to better fit the underlying CT data, avoid mesh intersections, and enforce local developability. To do this, we set up several constraints that apply forces to vertices in the mesh. We use the corresponding position of each vertex in E_2 to inform constraints applied in E_3. We forward integrate the system by a fixed number of time steps after each round of vertex additions and update vertex positions in E_3. After each integration step, we compute a mesh normal ($\hat{\mathbf{m}}$) for each vertex in E_3 based on the positions of its 1-ring, edge-connected neighbors.

We apply a **gradient force** to each vertex using $\mathbf{r}_x$, $\mathbf{r}_y$, and $\mathbf{r}_z$, calculated in during segmentation. This force helps to direct vertices towards brighter regions in $\mathbf{R}$. We scale this gradient force by 0.001.

$$\mathbf{f} = 0.001 \begin{bmatrix} r_x \\ r_y \\ r_z \end{bmatrix} \tag{20}$$

We apply **repulsion constraints** between neighboring vertices in E_3 in order to enforce paper layer separation. We construct two cones with height w_{exp} and base radius $c_{cone} = 2$ voxels along the positive and negative mesh normals of each vertex.

For two vertices i and j with displacement $\mathbf{d}_{j,i} = \mathbf{e_{3,i}} - \mathbf{e_{3,j}}$, vertex i is in the cones oriented along $\hat{\mathbf{m}}_j$ if:

$$0 \leq \mathbf{d}_{j,i} \cdot \hat{\mathbf{m}}_j \leq w_{exp} \tag{21}$$

$$\|\mathbf{d}_{j,i} - (\mathbf{d}_{j,i} \cdot \hat{\mathbf{m}}_j)\hat{\mathbf{m}}_j\| < \frac{c_{cone}(\mathbf{d}_{j,i} \cdot \hat{\mathbf{m}}_j)}{w_{exp}} \tag{22}$$

We calculate the forces on vertices i and j due to the interaction with the cones around vertex j:

$$\begin{aligned} \mathbf{f}_i &= \begin{cases} \frac{\mathbf{e_{3,i}} - \mathbf{e_{3,j}}}{\|\mathbf{e_{3,i}} - \mathbf{e_{3,j}}\|^2} & \text{if inside cones and } \|\mathbf{e_{2,i}} - \mathbf{e_{2,j}}\| > 20 \\ \frac{\mathbf{e_{3,i}} - \mathbf{e_{3,j}}}{\|\mathbf{e_{3,i}} - \mathbf{e_{3,j}}\|^2} & \text{if inside cones and } \|\mathbf{e_{2,i}} - \mathbf{e_{2,j}}\| \text{ is undefined and } \mathbf{d}_{j,i} \cdot \hat{\mathbf{m}}_j > 0.5 w_{exp} \\ \mathbf{0} & \text{otherwise} \end{cases} \\ \mathbf{f}_j &= -\mathbf{f}_i \end{aligned} \tag{23}$$

We omit repulsion forces between vertices that are close together (within 20 voxels) in E_2. $\|\mathbf{e_{2,j}} - \mathbf{e_{2,i}}\|$ is undefined when i and j are not part of the same 2D embedding (if $\mathbf{e_{2,i}} \in E_{2,n}$ and $\mathbf{e_{2,j}} \notin E_{2,n}$), or if one or both of the points have yet to be mapped to E_2. In these cases, we always apply repulsion forces.

We apply **smoothing constraints** between neighboring vertices in E_3. We construct a cylinder with height w_{exp} and base radius $c_{cyl} = 3$ voxels centered at each vertex, oriented along its mesh normal $\hat{\mathbf{m}}_i$.

For two vertices i and j with displacement $\mathbf{d}_{j,i} = \mathbf{e_{3,i}} - \mathbf{e_{3,j}}$, vertex i is in the cylinder oriented along $\hat{\mathbf{m}}_j$ if:

$$0 \leq \mathbf{d}_{j,i} \cdot \hat{\mathbf{m}}_j \leq \frac{w_{exp}}{2} \tag{24}$$

$$\|\mathbf{d}_{j,i} - (\mathbf{d}_{j,i} \cdot \hat{\mathbf{m}}_j)\hat{\mathbf{m}}_j\| < c_{cyl} \tag{25}$$

We calculate the forces on vertices i and j due to the interaction with the cylinder around vertex j:

$$\begin{aligned} \mathbf{f}_i &= \begin{cases} \frac{\text{sign}((\mathbf{e_{3,i}} - \mathbf{a}_j) \cdot \hat{\mathbf{m}}_j)}{\|\mathbf{e_{3,i}} - \mathbf{e_{3,j}}\|^2} \hat{\mathbf{m}}_j & \text{if inside cylinder and } (\|\mathbf{e_{2,j}} - \mathbf{e_{2,i}}\| < 50 \text{ voxels or undefined}) \\ \mathbf{0} & \text{otherwise} \end{cases} \\ \mathbf{f}_j &= -\mathbf{f}_i \end{aligned} \tag{26}$$

We apply a smoothing constraint when $\|\mathbf{e_{2,j}} - \mathbf{e_{2,i}}\|$ is undefined to help align separate mesh components as they grow toward each other to encourage merging (described below).

We enforce **distance constraints** along mesh edges in E_3. The nominal length (d_0) of a distance constraint in $\mathcal{A}$ is given by the initial length of the edge when it is first formed. Additionally, we calculate $\mathbf{d}_{i,j}$ based on vertex positions in E_3.

We forward integrate the net forces on each vertex, and apply viscous damping to help the system reach convergence.

As mesh propagation proceeds, we identify mesh components whose boundaries begin to approach each other in $\mathcal{A}$. For each vertex i on the boundary of a component in $\mathcal{A}$, we identify any vertices j that satisfy the following requirements:

- $\mathbf{e_{2,i}} \in E_{2,n}$ and $\mathbf{e_{2,j}} \in E_{2,m}$, but $E_{2,n} \neq E_{2,m}$
- 0.5 voxels $< \|\mathbf{e_{3,i}} - \mathbf{e_{3,j}}\| <$ 3 voxels

- $\hat{\mathbf{m}}_i$ and $\hat{\mathbf{m}}_j$ are aligned within $^{\pi}/_{6}$ radians

We identify pairs of components in E_3 with large, interconnected groups of vertices ($> c_{merging} = 200$) that satisfy the above requirements. For each pair of components in E_3, we compute a rigid transformation to bring their 2D embeddings ($E_{2,n}$ and $E_{2,m}$) into the same reference frame. The transformation from $E_{2,m}$ to $E_{2,n}$ may include a translation, rotation, and reflection.

First we incrementally add several vertices from $E_{2,m}$ to $E_{2,n}$ using the process described in the previous section. Using these vertices as a reference, we compute an optimal (least-squares) rigid transformation from $E_{2,m}$ to $E_{2,n}$ via Singular Value Decomposition (SVD) [**9**, **10**]. Once we've added all points from $E_{2,m}$ to $E_{2,n}$, we connect the mesh components with edges in both E_3 and $E_{2,n}$.

We found that enforcing **growth concurrency** between adjacent layers tends to increase the quality of the mesh growing results. We do this through a volumetric approach. We construct a growth concurrency volume at the resolution of the original XMT scan from the flagged points from the segmentation step. For each of these points, we calculate a strip of voxels along $\hat{\mathbf{n}}$, corresponding to the calculated width of the paper at that point. We mark all voxels in this strip as part of the concurrency volume. As we add new vertices to the boundary of the mesh, growth concurrency is enforced by applying an additional constraint: the voxel containing $e_{3,projected}$ must not be part of the concurrency volume. As new points are added to the mesh, we eliminate any nearby flagged points and recalculate the concurrency volume. At times the growth of some regions of the mesh may be stalled to allow a nearby region to catch up.

Texturing

We construct a 2D grid of pixels (t user-defined resolution) from the bounding box of the E_2. We identify the three closest mesh vertices (with 2D positions $\mathbf{e_{2,i}}$, $\mathbf{e_{2,j}}$, and $\mathbf{e_{2,k}}$ and 3D positions $\mathbf{e_{3,i}}$, $\mathbf{e_{3,j}}$, and $\mathbf{e_{3,k}}$) to the center of each pixel ($\mathbf{e_{2,pixel}}$) in the grid. We calculate the barycentric coordinates (u, v, w)[**11**] of $\mathbf{e_{2,pixel}}$ relative to the three vertices and use these coordinates to calculate a subvoxel position for $\mathbf{e_{3,pixel}}$.

We calculate a greyscale value at $\mathbf{e_{3,pixel}}$ using trilinear interpolation of $\mathbf{R}$. After repeating this process for each pixel in the 2D bitmap grid, the surface of the paper — and the text it carries with it — is revealed.

Using computed mesh normals, we can **offset** the previously grayscale texture by a small distance ϵ in either direction. We retrieve the mesh normals ($\hat{\mathbf{m}}_1$, $\hat{\mathbf{m}}_2$, and $\hat{\mathbf{m}}_3$) of vertices i, j, and k. Then, we use the barycentric coordinates to calculate an interpolated mesh normal $\hat{\mathbf{m}}_{pixel}$:

$$\hat{\mathbf{m}}_{pixel} = u\hat{\mathbf{m}}_i + v\hat{\mathbf{m}}_j + w\hat{\mathbf{m}}_k \tag{27}$$

We calculate an offset ϵ from $\mathbf{e_{3,pixel}}$:

$$\mathbf{e}^*_{\mathbf{3,pixel}} = \mathbf{e_{3,pixel}} + \epsilon\hat{\mathbf{m}}_{pixel} \tag{28}$$

We analyze the curvature of the 3D mesh to construct a pseudo-color image that reveals the **crease pattern** of the folded letter packet. We use a red color to represent mountain folds, and a blue color to represent valley folds. The opacity of the color represents the relative curvature, which allows users to differentiate between sharp folds and other slight deformations of the paper.

We calculate the curvature of each edge (c_{edge}) with vertices i and j in by:

$$c_{edge} = \frac{(\hat{\mathbf{m}}_i - \hat{\mathbf{m}}_j) \cdot (\mathbf{e_{3,i}} - \mathbf{e_{3,j}})}{\|\mathbf{e_{3,i}} - \mathbf{e_{3,j}}\|^2} \tag{29}$$

We estimate the curvature at each mesh vertex by taking the geometric mean of the curvature of the virtual "edges" between each vertex ($\mathbf{e_{3,center}}$) and its exclusive 10-ring neighbors ($\mathbf{e_{3,neighbor}}$):

$$c_{center} = \sum_{\substack{10-ring \\ neighbors}} \frac{(\theta_{right} + \theta_{left})}{2} \left[\frac{(\mathbf{m_{neighbor}} - \mathbf{m_{center}}) \cdot (\mathbf{e_{3,neighbor}} - \mathbf{e_{3,center}})}{\|\mathbf{e_{3,neighbor}} - \mathbf{e_{3,center}}\|^2} \right] \tag{30}$$

We use a 10-ring neighborhood to decrease noise in the calculation.

The sign of c indicates whether the vertex is located on a mountain or valley fold. The magnitude of c corresponds to the fold angle of the crease. We compute the curvature at a given pixel (c_{pixel}) by barycentric coordinates of neighboring vertices i, j, and k:

$$c_{pixel} = uc_i + vc_j + wc_k \tag{31}$$

Pixels with negative c_{pixel} are colored blue, while pixels with positive c_{pixel} are colored red. The opacity of the pixel is determined by the magnitude of c_{pixel}.

We clamp the opacity to the range [0,255] and draw the pixels on a white background so that flat regions of the mesh appear white.

Supplementary References

[1] R. Ahrendt, D. Van der Linden, The postmasters' piggy bank: Experiencing the accidental archive. *Fr. Hist. Stud.* **40(2)**, 189–213 (2017).

[2] R. Ahrendt, N. Akkerman, J. Dambrogio, D. S. Smith, and D. van der Linden. The Brienne Collection, *Early Modern Letters Online*, emlo.bodleian.ox.ac.uk (2017).

[3] A. N. Z. Evershed, D. Mills, G. Davis. Multi-species beam hardening calibration device for X-ray microtomography. *Proc. SPIE* **8506** (2012).

[4] L. A. Feldkamp, L. C. Davis, J. W. Kress, Practical cone-beam algorithm. *J. Opt. Soc. Am. A.* **1**, 612–619 (1984).

[5] G. R. Davis, A. N. Z. Evershed, D. Mills, Quantitative high contrast X-ray microtomography for dental research. *J. Dent.* **41(5)**, 475–482 (2013).

[6] C. Steger, An unbiased detector of curvilinear structures. *IEEE T. Pattern Anal.* **20.2**, 113–125 (1998a).

[7] C. Steger, "Unbiased extraction of curvilinear structures from 2D and 3D Images," thesis, Technical University of Munich (TUM) (1998b).

[8] T. Wagner and M. Hiner. "Ridge (Line) Detection Plugin (Fiji)". In: https://imagej.net/Ridge Detection (2017).

[9] K. S. Arun, T. S. Huang, S. D. Blostein, Least-squares fitting of two 3-D point sets. *IEEE T. Pattern Anal. PAMI-9.5* **5**, 698–700 (1987).

[10] O. Sorkine-Hornung, M. Rabinovich, "Least-squares rigid motion using SVD" (ETH Zurich, 2017).

[11] C. Ericson, *Real-time collision detection* (Elsevier, San Francisco, 2004), pp. 46-48.
