# Supplementary material for: Unlocking history through automated virtual unfolding of sealed documents imaged by X-ray microtomography
Source: Nat Commun. 2021 Mar 2;12:1184. doi: 10.1038/s41467-021-21326-w (PMC7925573; doi:10.1038/s41467-021-21326-w)
Supplement: Supplementary file 5 — Supplementary Data [file 41467_2021_21326_MOESM5_ESM.zip › 248751_3_supp_5110867_qk9r2l.pdf]

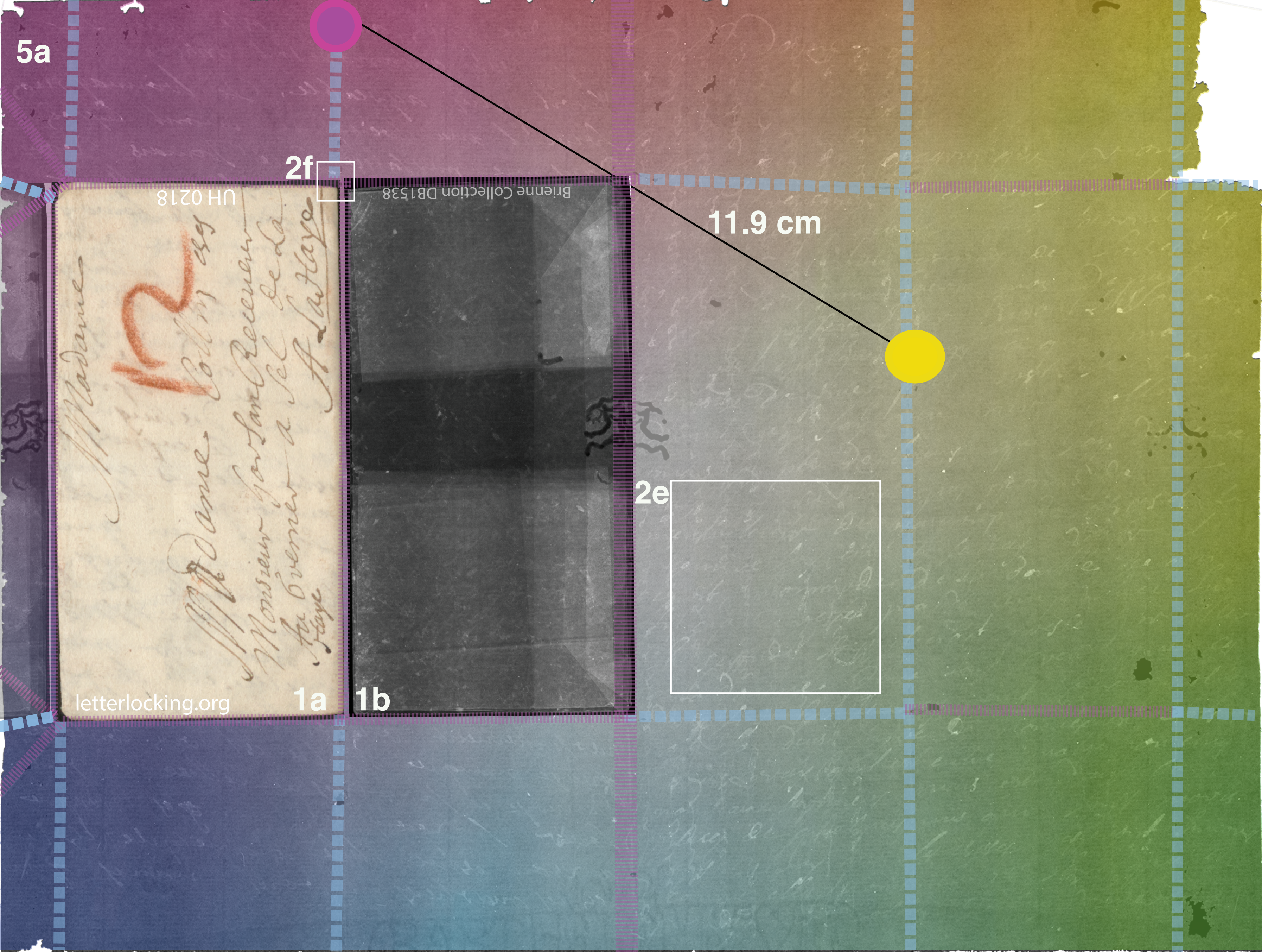
5a
2f
UH 0218
Brienne Collection DB1538
11.9 cm
2e
letterlocking.org
1a
1b

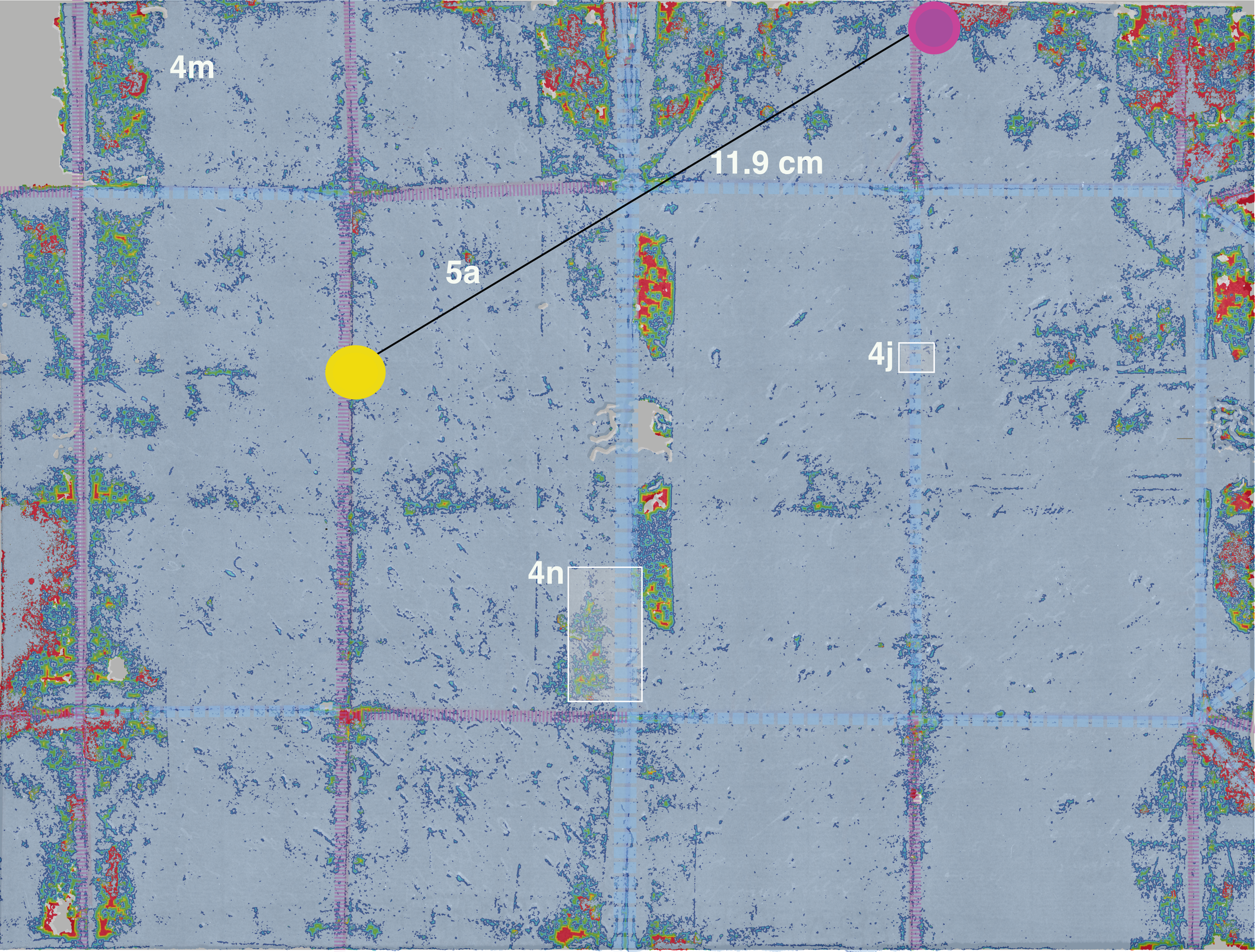
4m
11.9 cm
5a
4j
4n
